# Ureteral access sheath or percutaneous nephrostomy during flexible ureteroscopy: which is better?

**DOI:** 10.1007/s00240-024-01683-z

**Published:** 2025-01-03

**Authors:** Mohamed Abdelrahman Alhefnawy, Moaz Fathy Ismail Abdelrahman, Hosam Abdel-fattah Abo-Elnasr, Helmy Ahmed Eldib

**Affiliations:** 1 Urology Department, Benha University, Benha, Qalubia Egypt; 2 Urology Department, Royal Blackburn Teaching Hospital, East Lancashire Hospitals NHS Trust, Blackburn, UK

**Keywords:** Ureter, Ureteroscopy, Percutaneous nephrostomy, Flexible, Access sheath

## Abstract

Studies in literature discussed the drawbacks of the ureteral access sheath use in flexible ureteroscopy and in the same time mentioned the benefits of ureteral access sheath in decreasing the incidence of urosepsis and better stone free rate. In the current study we aim to compare between percutaneous nephrostomy tube (PCN) insertion before flexible ureteroscopy and conventional ureteral access sheath (UAS) flexible ureteroscopy in terms of safety, efficacy and perioperative outcomes. In all, 100 Patients aged 20 to 67 years with upper ureteric stones and mild hydronephrosis or renal pelvic stones less than 20 mm with mild hydronephrosis were randomized into 2 groups; patients undergoing PCN insertion before flexible ureteroscopy, and patients undergoing the conventional UAS flexible ureteroscopy. Patients with active urinary tract infection, patients with urinary diversions or malformations and patients with uncontrolled coagulable status were excluded from the study. Perioperative data were recorded. This study was conducted on 50 PCN group and 50 UAS group. Age varied from 20.0 to 67.0 years. Males consisted more than half of study groups, 52% of PCN group and 66% of UAS group. Weak significant difference was found in need for ureteral pre-operative stenting between groups (8% with PCN vs. 22% with UAS, p 0.04995). There was no significant difference between two groups in intra-operative complications (mucosal injury, failed operation, perforation, false passage and conversion to other procedure), but there was significant difference in bleeding between the groups (6% with PCN vs. 22% with UAS, *p* = 0.021). There was no significant difference between two groups in post-operative complications (infection, fever, pain, hematuria, other complications, stone free rate, readmission and stent duration), but there was significant decrease in operative time (48.85 ± 13.861 in PCN group versus 56.82 ± 14.61 in UAS group, *p* = 0.0003). We conclude that PCN insertion before flexible ureteroscopy provides a safe technique with comparable outcomes to UAS use.

## Introduction

Retrograde intra renal surgery (RIRS) is continuously evolving and its role in treating upper urinary tract stones is increasing with better outcomes and less complication rates as compared with extracorporeal shockwave lithotripsy and percutaneous nephrolithotomy [1, 2].

Ureteric Access Sheath (UAS) has multiple benefits with its usage in endourological procedures, so it is commonly used during flexible ureteroscopy (FURS) minimizing scope damage remarkably [3], facilitating multiple passage in the kidney with better vision due to improved irrigation and outflow, also decreasing intra renal pressure [4, 5]. There is discordant data about the role of UAS in decreasing the risk of urinary tract infection and sepsis [6, 7].

Moreover, the UAS has an added merit of passage of small stone particles formed during laser lithotripsy with the irrigation fluid exit. Nevertheless, usage of UAS could be harmful to the ureter either due to its over distention affecting blood flow of the ureter and may cause ureteral stricture or direct trauma to the ureter during sheath insertion [8]. UAS has different sizes and lengths for clinical use but the choice of the appropriate size depends on the urologist preference and the anatomy of the ureter regarding its compliance or previous stenting [9].

Percutaneous nephrostomy (PCN) is the gold standard for treatment of post renal obstruction as a urinary diversion or as an initial step of another urological procedure with high success and low complication rates [10, 11]. PCN allow the urologist to get access to the renal collecting system without injection of contrast material intravenously, so it is the most reliable procedure expending less time and material and it is largely employed in the management of urolithiasis in many urological centers [12, 13].

Therefore, we hypothesize that ultrasound guided PCN insertion in lower calyx before flexible ureteroscopy despite its proposed invasiveness will get the benefits of UAS in terms of stone free rate (SFR) and reduced intra-renal renal pressure. Additionally it presents a less invasive alternative to UAS with its documented ureteral complications.

## Patients and methods

Our prospective randomized study was conducted at benha university hospitals on adult patients aged 20 to 67 years with renal pelvic or calyceal stones ranging from 14 mm to 27 mm comparing usage of ureteral access sheath and percutaneous nephrostomy during flexible ureteroscopy, patients were randomly assigned to one of the two groups. We used closed envelope method for randomizing patients blindly to any of the two groups. Exclusion criteria were patients with active urinary tract infection, bilateral stones, patients with urinary diversions or malformations and patients with uncontrolled coagulable status. An informed consent was obtained from all patients. This study was conducted according to ethical principles stated in the Declaration of Helsinki (2013) [7] and the requirement of faculty of medicine, Benha university.

Patients’ demographics regarding age, gender, body mass index and comorbidities were recorded. History, physical examination and laboratory investigations were done to exclude active urinary tract infection. Non- contrast computed tomography of the abdomen and plain abdominal radiography, stone side, stone burden, number of stones, hydronephrosis with its grade and Hounsfield Unit were recorded.

### Operative technique

Patients were classified into two groups according to technique of operation:

#### Group A: flexible ureteroscopy and UAS

Patients were placed in the lithotomy position under general anesthesia and given appropriate antibiotic cover according to previous culture and sensitivity. The bladder and ureteric orifice were directly visualized by rigid cystoscopy prior to the introduction of a safety guide wire., a semi rigid ureteroscopy was performed over a second working wire up to the pelvi-ureteric junction (PUJ) or as far proximally as safely achievable, allowing passive dilatation of the ureteric orifice and ureter. Ureteral dilation using serial dilators was carried out if needed and up to 14 French (Fr). Two sizes of UAS were used: COOK™ Medical Flexor 12/14 French (Fr) (wider) and 9.5/11.5Fr (narrower) according to the degree of permitted ureteral dilatation and size of the stone, the need for its use was according to the surgeon estimation on case-by-case basis. Appropriately, sized UAS was then inserted over the wire and positioned just distal to the PUJ or below upper ureteric stone under fluoroscopic guidance. We then performed a flexible ureteroscopy using Olympus™ URF-V2 (8.5 Fr) flexible ureteroscope to inspect the proximal ureter if there is upper ureteric stone, renal pelvis, and calyces for the presence of stones. Irrigation with 0.9% saline solution via a pressure-infusing system. Next, Holmium laser lithotripsy with a holmium: YAG laser [20 W; Lumenis™ (UK) Ltd., Elstree™, UK] using a 272-micron laser fiber (Lumenis™, Inc.) was carried out and/or stone extraction with a basket device.

#### Group B: flexible ureteroscopy without UAS and insertion of PCN

Under general anesthesia in the supine position and given appropriate antibiotic according to previous culture and sensitivity, ultrasound guided 8 Fr PCN was inserted trans papillary in the lower calyx of the kidney by an expert intervention radiologist. This narrow caliber of PCN did not lower intrarenal pressure significantly to the degree of obscuring visual field and it did not obstruct the field nor interfered with the lithotripsy process due to the narrow caliber of the nephrostomy tube as this narrow caliber does not permit exit of irrigating fluid at a rate faster than its inflow and the fact that we used pressurized saline bag kept optimal surgical field. Additionally, whenever we felt that the nephrostomy tube was advanced in the pelvicalyceal system to the degree of obstructing the field or interfering with the lithotripsy procedure it was withdrawn back cautiously under vision of the flexible ureteroscope till just its tip is in the pelvicalyceal system it was then secured to the skin.

The bladder and ureteric orifice was directly visualized by rigid cystoscopy prior to the introduction of a safety guidewire., a semi rigid ureteroscopy was performed over a second working wire up to the pelviureteric junction (PUJ) or as far proximally as safely achievable, allowing passive dilatation of the ureteric orifice and ureter. Ureteral dilation was done using serial dilators up to 10 Fr if needed. We then performed a flexible ureteroscopy using Olympus™ URF-V2 (8.5 Fr) flexible ureteroscope and completed the procedure as described in the previous group. Nephrostomy was open for irrigation and for small dusted stone particles to get out through it. At the end of the procedure, a double pigtail ureteral stent was left for 14 days. Once the operation is finished, PCN was closed for 2 h to ensure that the renal unit is properly drained by the stent then if no pain or sockage were observed, the PCN catheter was removed.

In both groups, the same settings of pressurized saline bag irrigation system and the same laser settings with combined fragmentation and dusting techniques were used.

### Postoperative assessment

Operative time, intra-operative complications (bleeding, perforation, incompletion of procedure) and hospitalization periods were recorded. Ureteral complications were graded according to the modified clavien dindo score [14].

In PCN group, operative time was calculated by adding time used in PCN insertion including the time of changing the patient position from prone to supine for subsequent endoscopic treatment to the time used during the procedure itself.

Non- contrast computed tomography with 2–3 mm cuts was done in all cases in the first month after the operation to assess stone-free rates and ureteral stricture formation. According to non-contrast CT findings, Stone-free data were classified into three grades: Grade A (no stones on CT scan), absolute stone free, Grade B (< /= 2 mm fragments) relative stone free, and Grade C (2.1–4 mm) fragments relative stone-free. Evaluation for success was done at 1 month and 3 months after the procedure.

Criteria of postoperative stenting were presence of ureteral trauma and residual stones.

### Outcomes

The primary outcome was the success rate presented by the stone free rate and operative time. The secondary outcomes were complications including ureteral complications, infectious complications and gross hematuria.

### Sample size calculation

Assuming that success rate of PCN group was 95.1% (the test group) and 82% in the control group (traditional technique without PCN) after applying continuity correction, the study would require a sample size of: 50 for each group (i.e. a total sample size of 100, assuming equal group sizes), to achieve a power of 90% and a level of significance of 5%, drop-out rate of 10%, for declaring that the PCN is not inferior to the traditional technique at a -10% margin of non-inferiority (assuming that a larger proportion is desirable).

### Statistical analysis

Data were expressed as means ± Standard deviation for parametric data and median, Minimum and maximum for non-parametric data. Tests of significance used were: Test of Kolmogorov- Smirnov and Shapiro Wilk were used to test normality of distribution of numerical variables. Pearson Chi-square (χ2) was used as tests of significance of association between two categories. Whenever more than 20% of expected values were less than 5, Fisher Exact test (FEX) was used instead. Independent t test was used to test significance difference between two groups for parametric quantitative variables and Mann Whitney test (U) for non-parametric variables. Binary logistic Regression analysis is used with odds ratio (OR) expression and its 95%confidence interval (95%CI) to estimate the relationships between a binary categorical dependent variable (outcome) and other independent variables (predictors), while linear regression used in case of numerical dependent variable. Univarate analysis was done first, then only significant predictors were included into the multivariante regression model. The level of significance of our data was 95%, so, *p* value > 0.05 was considered a non-statistically significant difference, while *p* value < 0.05 was considered a statistically significant difference.

## Results

This study was conducted on 50 PCN group and 50 UAS group. Age ranged from 20.0 to 67.0 years. Males consisted more than half of study groups, 52% of PCN group and 66% of UAS group (Table 1).

**Table 1:** Demographics and medical history preoperative data

Variable	Total (n = 100)		PCN group (*n* = 50)		UAS group (n = 50)		*P* value
**Demographic data**							
**Age (Years)**							
Mean ± SD	40.22 ± 9.67		40.70 ± 9.069		39.51 ± 10.282		
Median	39.5		41		39		0.622
Min-Max	20.0–67.0		24.0–62.0		20–67		
**Sex**	**N**	**%**	**N**	**%**	**N**	**%**	
Male	59	59	26	52	33	66	0.155
Female	41	41	24	48	17	34	
**Medical History**							
**Medical History**							
Free	88	88	44	88	44	88	
HTN	6	6	3	6	3	6	1
NIDDM	5	5	3	6	2	4	
HTN and DM	1	1	0	0	1	2	
**Surgical History**							
Free	67	67	36	72	31	62	
PCNL	3	3	1	2	2	4	0.733
Pyelolithotomy	3	3	1	2	2	4	
SWL	17	17	9	18	8	16	
URS	9	9	3	6	6	12	
URS and SWL	1	1	0	0	1	2	

Data are expressed as mean ± SD, median (Min-Max), number and percentages, used tests of significance: Independent T test, Chi square and Fisher Exact tests, significance level at *P* < 0.05

There was significant difference in distribution of stone free rate between both groups as PCN group showed higher grade A (absolute free rate) and minimal grade C (2.1 to 4 mm stone residuals) compared to UAS group, (96% versus 90%, and 0% versus 10%), *p* = 0.028*). Additionally, highly significant decrease in operative time was observed (48.85 ± 13.861 min in PCN group versus 56.82 ± 14.61 min in UAS group, *p* = 0.0003*).

Weak significant difference was found in need for ureteral pre-operative stenting between both groups (22% with UAS vs. 8% with PCN, p 0.04995), while, there was highly significant increased need for post-operative stenting in UAS group compared to PCN group, (86% with PCN vs. 48% with PCN, *p* < 0.001).

There was no significant difference of age and sex between study groups, *p* = 0.622 and 0.155, respectively. There was no significant difference between two groups in stone characters (number of stones, stone side, size, location and Hounsfield unite), *p* = 0.307, 0.317, 0.524, 0.728 and 0.944, respectively (Table 2).

**Table 2:** Distribution of preoperative data in study groups

Variable	Total (n = 100)		**PCN group (n = 50)**		UAS group (n = 50)		*P* value
	**N**	%	**N**	%	N	%	
**Main presentation**							
**Loin Pain**	99	99	49	98	50	100	0.5
**Hematuria**	19	19	9	18	10	20	0.799
**Urine analysis**							
**Pus cells**							
0–5	68	68	38	76	30	60	
-10	4	4	0	0	4	8	0.063
-50	25	25	12	24	13	26	
-100	2	2	0	0	2	4	
- over 100	1	1	0	0	1	2	
**Urine culture(n = 26)**							
E coli	15	15	8	16	7	14	
Klebseilla	2	2	1	2	1	2	0.405
Staphylococcusaureus	3	3	0	0	3	6	
No growth	80	80	41	82	39	78	
**Kidney function tests**							
**Blood Urea (mg/dl)**							
Mean ± SD	33.71 ± 7.836		35.72 ± 7.68		31.76 ± 7.609		0.012*
Median	33		35		30		
Min-Max	18.0–54.0		24.0–54.0		18.0–46.0		
**Serum creatinine (mg/dl)**							
Mean ± SD	1.012 ± 0.224		0.986 ± 0.184		1.039 ± 0.259		0.457
Median	1		1		1.04		
Min-Max	0.50–1.90		0.6–1.4		0.5–1.90		
**Stone character**							
**Number of stone**							
One stone	96	96	47	94	49	98	0.307
More than one	4	4	3	6	1	2	
**Stone Side**							
Left	51	51	28	56	23	46	0.317
Right	49	9	22	44	27	54	
**Stone Size (mm)**							
Mean ± SD	22.33 ± 2.648		22.16 ± 3.00		22.50 ± 2.26.0		
Median	23		23		23		0.524
Range	14.0–27.0		14.0–27.0		15.0–27.0		
**Stone Location**							
Pelvis	84	84	41	82	43	86	0.585
Lower calyx	15	15	9	18	6	12	0.401
Middle calyx	14	14	7	14	7	14	1
Upper calyx	16	16	9	18	7	14	0.585
**Hounsfield unite**							
Mean ± SD	963.64 ± 322.78		962.06 ± 305.69		965.22 ± 342.12		0.944
Median	978		978		978		
Range	250.0-1700.0		450.0-1700.0		250.0-1700.0		

Data are expressed as mean ± SD, median (Min-Max), number and percentages, used tests of significance: Fisher Exact, chi square, Mann Whitney and Independent T tests, *: significant at *P* < 0.05

There was no significant difference between two groups in intra-operative complications (mucosal injury, failed operation, perforation, false passage and conversion to other procedure), but there was significant difference in bleeding between the groups (6% with PCN vs. 22% with UAS, *p* = 0.021).

There was no significant difference between two groups in post-operative complications (infection, fever, pain, hematuria, other complications, readmission and stent duration) (Table 3).

**Table 3:** Distribution of perioperative data in study groups

Variable	Total (n = 100)		**PCN group (n = 50)**		UAS group (n = 50)		*P* value
	**N**	%	**N**	%	N	%	
**Stenting**							
**Pre-operative stenting**	15	15	4	8	11	22	0.04995
**Post-operative stenting**	67	67	24	48	43	86	< 0.001**
**Intra-operative complication**							
**Mucosal injury**	9	9	3	6	6	12	0.243
**Bleeding (Active gross hematuria)**	14	14	3	6	11	22	0.021*
**Post-operative hemoglobin**	13.02 ± 1.33		13.29 ± 1.18		12.74 ± 1.43		0.064
	13.2		13.35		13		
	9.0-14.9		10.0-14.9		9.0-14.8		
Failed***	4	4	1	2	3	6	0.307
**Perforation**	1	1	1	2	0	0	0.315
**False passage**	4	4	1	2	3	6	0.307
Converted to other operation****	2	2	0	0	2	4	0.153
**Post-operative complication**							
**Febrile UTI**	8	8	2	4	6	12	0.14
**Pain**	38	38	16	32	22	44	0.216
**Hematuria**	22	22	9	18	13	26	0.334
**Other Complications**	2	2	0	0	2	4	0.153
**Stone free rate**							
Grade A: absolute free	93	94	48	96	45	90	0.028*
Grade B: (≤ 2 mm)	2	2	2	4	0	0	
Grade c: (2.1 to 4mm)	5	2	0	0	5	10	
Grade d: < 4 mm	0	0	0	0	0	0	
**Readmission**	2	2	0	0	2	4	0.153
**Operative time (min)**							
Mean ± SD	52.83 ± 14.78		48.85 ± 13.861		56.82 ± 14.61		0.000262**
Median	60		50		60		
Range	18.0–75.0		20.0–70.0		18.0–75.0		
**Stent duration(days)**							
Mean ± SD	24.57 ± 5.096		25.56 ± 4.845		23.57 ± 5.248		0.105
Median	25		25		22		
Range	15.0–40.0		15.0–40.0		15.0–30.0		

Data are expressed as mean ± SD, median (Min-Max), number and percentages, used tests of significance: chi square, Fisher Exact and Mann Whitney tests, *: significant at *P* < 0.05, **: Highly significant at *P* < 0.01

*** fail: include cases of encountered ureteric stricture, pyuria and active hematuria obscuring surgical field, in these cases only stenting with JJ was done

**** converted to other procedure: namely PCNL

Univariate binary logistic regression analysis was performed to weigh the risk of different variables as predictors of risk of conversion to other procedure. Only number of stones was statistically significant in the logistic model, presence of more than one stone increases the risk of conversion to other procedure by 31.667 folds more than in case of one stone. All selected variables were not statistically significant predictors. These results suggest that these factors may need further investigations on larger samples to detect their efficacy as potential risk factors of conversion into other procedure.

Univariate binary logistic regression analysis was performed to weigh the risk of different variables as predictors of risk of post-operative infection. All selected variables were not statistically significant predictors. These results suggest that these factors may need further investigations on larger samples to detect their efficacy as potential risk factors of post-operative infection.

Univariate binary logistic regression analysis was performed to weigh the risk of different variables as predictors of risk of bleeding (Table 4). Only technique used was statistically significant in the logistic model, UAS technique increased the risk of bleeding by 4.419 folds more than PCN technique. All selected variables were not statistically significant predictors. These results suggest that these factors may need further investigations on larger samples to detect their efficacy as potential risk factors of bleeding.

**Table 4:** Logistic regression analysis for the different variables as risk factors of bleeding

Risk Factors	Univariate Analysis		Multivariate Analysis	
	OR (95% C.I)	Sig.	Sig.	OR (95% C.I)
Age (Years)	0.956(0.897–1.019)	0.163		
Gender, male	0.654(0.211–2.031)	0.462		
Number of stone	2.128(0.206–22.037)	0.527		
Stone Side, Left	1.886(0.584–6.089)	0.289		
Stone size	1.085(0.863–1.364)	0.486		
Positive Urine culture	1.107(0.278–4.411)	0.885		
Upper Ureter	1.167(0.235–5.794)	0.850		
Middle Ureter	0.973(0.193–4.899)	0.973		
Lower Ureter	2.528(0.306–20.912)	0.390		
Pelvis	0.857(0.183–4.257)	0.850		
Preoperative stent	1.068(0.214–5.340)	0.936		
Technique, UAS	4.419(1.151–16.966)	0.03*	0.03*	4.419(1.151–16.966)
Constant			0.001*	0.14
Model significance			0.018*	
Classification percentage			86%	
Pseudo r square			0.055	

OR: odds ratio, 95%(C.I.): 95% confidence interval, sig.: significant at *P* < 0.05

## Discussion

The use of ureteral access sheath in retrograde intra renal surgery has been associated with variable degrees of ureteric injury including: mucosal injury, urinary extravasation, ureteral wall perforation, ureteral avulsion and postoperative ureteric stricture as reported by many studies [15–17]. Other authors reported that performing flexible URS without ureteral access sheath carries the risk of increased intra renal pressure with resulting pyelo-venous reflux that leads to increased incidence of urosepsis and septic shock [18, 19].

To our knowledge, our research is the first prospective randomized study that assess safety and efficacy of flexible ureteroscopy without UAS but with PCN insertion in comparison with flexible ureteroscopy with ureteral access sheath.

In our study, urosepsis occurred in 2 cases (4%) in the PCN group versus 6 cases (12%) in the UAS group (p 0.140). This agrees with Cristallo et al., 2022 [19] who reported that though not statistically significant, FURS with UAS had slightly more UTI than without UAS (11.6% vs8.1% respectively, p 0.455) and they believe that only stone diameter and positive preoperative urine culture increased infection risk, with last one almost triplicating it. On the other hand other investigators such as Villa et al., 2023 [20] recorded fever (*n* = 52; 11.5%), sepsis (*n* = 10; 2.2%), and septic shock (*n* = 6; 1.3%). Of those, UAS was not used in 29 (55.8%), 7 (70%), and 5 (83.3%) cases, respectively (all *p* > 0.05), however, on multivariable logistic regression analysis, performing URS without UAS was not associated with the risk of having fever and sepsis, but it increased the risk of septic shock (OR = 14.6; 95% CI = 1.08–197.1). This is consistent with the results of Yitgin et al., 2021 [15] who reported that postoperative febrile UTI was found in 5 (8%) of the patients without access sheath, and in 4 (8%) of the patients with access sheath (*p* = 0.733). The CROES’ research, the largest non-randomized prospective multicentric study published to date, showed that infectious complications, such as documented UTI or fever were more frequent when UAS was not employed (23.9% vs. 18.6% and 39.1% vs.28.6%, respectively) [22].

There are many other factors that contribute to increased incidence of urosepsis in flexible URS in general, including patient comorbidity, previous history of UTI, preoperative urine culture status, the presence of an indwelling DJ at the time of surgery, even with negative urine culture, and long operative time [23, 24]. In our study, univariate binary logistic regression analysis was performed to weigh the risk of different variables as predictors of risk of post-operative infection; all selected variables were not statistically significant predictors.

Increased incidence of urosepsis in cases done without UAS can be attributed to higher intra renal pressures resulting in pyelo venous and pyelo lymphatic reflux. Therefore, higher pressures are supposed to cause more infectious complications [25].

We believe in our study the insertion of ultrasound guided PCN in those cases done without UAS is the reason behind less incidence of urosepsis in this group as the PCN help draining irrigation fluid leading to decreased intra renal pressure during the procedure and consequently reducing the incidence of postoperative infectious complications.

In the current study, there was a statistically significant difference in stone free rate between both groups, the PCN group had a SFR of 96% versus 92% in the UAS group (p 0.047). This is consistent with Kourambas’ and Berquet’s studies who found that SFR was higher without UAS [26, 27]. This concurs with CROES study’s [22] SFR that was better without UAS (82.8% vs. 73.9%; *p* < 0.01). On the other hand, L’Esperance got better SFR using UAS (p 0.042) [28]. While, Yitgin et al., 2021 [15], reported that there was no significant difference in SFR between the two Groups and they attributed this to surgeon’s experience, operation technique performed with dusting and the ureteral JJ stent placement following the procedure.

We believe, in the study in our hands, the better SFR in the group without UAS is related to the role of PCN that helps flushing small stone particles out of the collecting system creating more clear vision and better stone clearance.

In our study, we found that the operative time in the PCN group was significantly less than the UAS group (p 0.000262). On Univariate linear regression analysis and multivariate linear model using operative time as the dependent variable we found that number of stones and technique used were statistically significant in the linear model, presence of more than one stone increases the risk of increasing operative time by 17.679 units more than in case of one stone, UAS technique increased risk of increasing operative time by 9.207 units more than PCN technique. This agrees with Yitgin et al., 2021 [15] and they believe the reason behind this was the inclusion of the time spent during UAS insertion and the need to return to the UAS with f-URS during initial insertion of the laser probe. Therefore, when UAS is not used, repeated entry and re-entry into the collecting system is not performed, and the surgeon completed the procedure with a single entry. CROES’ study [21] reported shorter operative time when UAS was not used (64.7 min vs. 80 min; *p* ≤ 0.01). This is consistent with Cristallo et al., 2022 [19] who reported that surgical times were reduced in non-UAS group (65 min without UAS vs. 90 min with; p 0.01). On the contrary, Kourambas et al. [25], reported faster surgeries (*p* < 0.05) when UAS was used.

Cristallo et al., 2022 [19] believe that UAS may speed up the procedure in non-expert hands; however, well-trained endourologists should not have any problems getting into the ureter with a flexible ureteroscope without UAS thus reducing costs and avoiding ureteral complications. However We disagree with their explanation as we believe that even in expert hands a big middle lobe increase the time needed to enter the ureteral orifice and difficult to maneuver into the upper urinary tract as is reduces the transmission of the movement from the urologist hands to the tip of the instrument.

As regard complications in this study, mucosal injury occurred in 3 cases (6%) in the PCN group versus 6 cases (12%) in the UAS group, bleeding (active gross hematuria obscuring surgical field) occurred in 3 cases (6%) in the PCN group versus 11 cases (22%) in the UAS group, we believe this can be attributed to the fact that an 8 fr nephrostomy was inserted by a professional intervention radiologist trans- papillary, under ultrasound guidance and its narrow caliber, on the contrary, UAS group had more bleeding due to ureteral trauma induced by its insertion and ureteral dilatation preceding its use, perforation occurred in 1 case (2%) in the PCN group, while, false passage occurred in 1 case (2%) in the PCN group versus 3 cases (6%) in the UAS group. This is in the same line with Traxer et al. 2013 [7] in their study, which examined 359 patients undergoing RIRS with 12/14 fr UAS, stated that the ureteral wall injury rate was 46.5%. Additionally, serious ureteral wall injury was detected in 13.3% of patients. They also reported that ureteral injury rate significantly decreased in patients who placed JJ-stents before RIRS.

Lildal et al. 2018 [28] compared RIRS patients with UAS used (10/12Fr) versus without UAS and found that ureteral injury rate was higher in patients with UAS usage (50% vs. 36%). Additionally, Lallas et al., 2002 [16] demonstrated that UAS was associated with a transient decrease in ureteral blood flow, which could potentially lead to further ureteral stricture.

As regard our study limitations, different surgeons’ experiences were not evaluated as a factor-affecting outcome of cases done without UAS. Randomizing patients in our study would be better if included other demographic aspects. The number of male in the UAS group is almost 2 times the number of female; this might be a cause of having more blood from prostatic urethra or from urethra distal to the urinary sphincter. More studies with larger sample size are needed to assess the safety and efficacy of PCN without UAS technique and its effect on patients postoperative QOL score, to assess the safety and efficacy of different sizes of UAS versus PCN without UAS and to correlate between the increased incidence of urosepsis and the use of narrow UAS where the gap between the sheath and the f-URS is only 1 Fr. Additionally, longer follow up periods are needed to detect long-term complications such as ureteral stricture secondary to the procedure.

## Conclusion

Our suggested technique (ultrasound guided PCN insertion in lower calyx before flexible ureteroscopy) demonstrates superior outcomes in terms of the main outcome of our study, such as a higher success rate (SFR) and fewer complications, particularly reduced bleeding with shorter operative time compared to using ureteral access sheath. Notably, these improvements are achieved without a significant increase in other complications.

## Data Availability

Research data are available upon request.
